# Machine-learning vs. logistic regression for preoperative prediction of medical morbidity after fast-track hip and knee arthroplasty—a comparative study

**DOI:** 10.1186/s12871-023-02354-z

**Published:** 2023-11-29

**Authors:** Christian Michelsen, Christoffer C. Jørgensen, Mathias Heltberg, Mogens H. Jensen, Alessandra Lucchetti, Pelle B. Petersen, Troels Petersen, Henrik Kehlet, The Center for Fast-track Hip Knee Replacement Collaborative group, Frank Madsen, Torben B. Hansen, Kirill Gromov, Thomas Jakobsen, Claus Varnum, Soren Overgaard, Mikkel Rathsach, Lars Hansen

**Affiliations:** 1 The Niels Bohr Institute, University of Copenhagen, Blegdamsvej 17, 2100 Copenhagen, Denmark; 2 Department of Anesthesia and Intensive Care, Hospital of Northern Zealand, Dyrehavevej 29 3400, Hillerød, Denmark; 3 The Centre for Fast-Track Hip and Knee Replacement, 7621, Rigshospitalet, Blegdamsvej 9, 2100 Copenhagen, Denmark; 4 Section of Surgical Pathophysiology, 7621, Rigshospitalet, Blegdamsvej 9, 2100 Copenhagen, Denmark

**Keywords:** Machine learning, Risk assessment, Hip replacement, Knee replacement, Enhanced recovery after surgery, Perioperative care, Postoperative complications

## Abstract

**Background:**

Machine-learning models may improve prediction of length of stay (LOS) and morbidity after surgery. However, few studies include fast-track programs, and most rely on administrative coding with limited follow-up and information on perioperative care. This study investigates potential benefits of a machine-learning model for prediction of postoperative morbidity in fast-track total hip (THA) and knee arthroplasty (TKA).

**Methods:**

Cohort study in consecutive unselected primary THA/TKA between 2014–2017 from seven Danish centers with established fast-track protocols. Preoperative comorbidity and prescribed medication were recorded prospectively and information on length of stay and readmissions was obtained through the Danish National Patient Registry and medical records. We used a machine-learning model (Boosted Decision Trees) based on boosted decision trees with 33 preoperative variables for predicting “medical” morbidity leading to LOS > 4 days or 90-days readmissions and compared to a logistical regression model based on the same variables. We also evaluated two parsimonious models, using the ten most important variables in the full machine-learning and logistic regression models. Data collected between 2014–2016 (n:18,013) was used for model training and data from 2017 (n:3913) was used for testing.

Model performances were analyzed using precision, area under receiver operating (AUROC) and precision recall curves (AUPRC), as well as the Mathews Correlation Coefficient. Variable importance was analyzed using Shapley Additive Explanations values.

**Results:**

Using a threshold of 20% “risk-patients” (n:782), precision, AUROC and AUPRC were 13.6%, 76.3% and 15.5% vs. 12.4%, 74.7% and 15.6% for the machine-learning and logistic regression model, respectively. The parsimonious machine-learning model performed better than the full logistic regression model. Of the top ten variables, eight were shared between the machine-learning and logistic regression models, but with a considerable age-related variation in importance of specific types of medication.

**Conclusion:**

A machine-learning model using preoperative characteristics and prescriptions slightly improved identification of patients in high-risk of “medical” complications after fast-track THA and TKA compared to a logistic regression model. Such algorithms could help find a manageable population of patients who may benefit most from intensified perioperative care.

**Supplementary Information:**

The online version contains supplementary material available at 10.1186/s12871-023-02354-z.

## Introduction

Prediction of postoperative morbidity and requirement for hospitalization is important for planning of health care resources. With regards to the common surgical procedures of primary total hip (THA) and knee arthroplasty (TKA), the introduction of enhanced recovery or fast-track programs has led to a significant reduction of postoperative length of stay (length of stay) as well as morbidity and mortality [1–3]. However, despite such progress, a fraction of patients still have postoperative complications leading to prolonged length of stay or readmissions [1, 3, 4]. Consequently, to prioritize perioperative care, many efforts have been published to preoperatively predict length of stay and morbidity using traditional risk factors such as age, preoperative cardio-pulmonary disease, anemia, diabetes, frailty, etc. [4–8]. These efforts have been based on traditional statistical methods, most often multiple regression analyses, and essentially concluding that it is “better to be young and healthy than old and sick”. Consequently, despite being statistically significant, conventional risk-stratification based on such studies has had a relatively limited clinically relevant ability to predict and reduce potentially preventable morbidity and length of stay [4–8].

More recently, machine-learning methods have been introduced with success in several areas of healthcare and where preliminary data suggest them to improve surgical risk prediction compared to traditional risk calculation in certain anesthetic and surgical conditions [9, 10]. This is also the case in THA, TKA and uni-compartmental knee replacement, where several publications on machine-learning algorithms for prediction of length of stay [11, 12] complications [13], disability [14], potential outpatient setup [15], readmissions [16] or payment models [17], have shown promising predictive value compared to conventional statistical methods [18].

However, few papers have included fast-track programs, and most are based on large databases with the presence of risk factors and complications often relying on administrative coding with limited information on perioperative care, follow-up and discharge destination. In our previous study of 9512 THA and TKAs within a fully implemented fast-track protocol and including the above information, we did not find advantages of machine-learning methods compared to logistic regression in predicting a length of stay > 2 days [19]. However, this may have been due to data imbalance, lack of details on medication and the chosen outcome of length of stay of > 2 days which may not be directly related to preoperative patient characteristics [19]. Furthermore, medical complications resulting in prolonged admission or readmissions may be more clinically relevant than focusing on length of stay when attempting to identify a relevant patient population for future perioperative interventions [20]. Especially within well-established fast-track protocols where LOS is about 1 day [1]. Thus, the combination of modern evidence-based surgical fast-track protocols with machine-learning models remain promising as it may provide an improved and continually developing basis for identifying which patients may benefit from more extensive preoperative evaluation and postoperative medical care.

Consequently, we used a large consecutive cohort of patients undergoing fast-track total hip and knee replacement within a national public health-care system [1] to develop and test a new machine-learning model with an extended number of preoperative variables including information on dispensed reimbursed prescriptions [21], for preoperative prediction of “medical” complications with prolonged length of stay or readmissions.

Our hypothesis was that these changes with regards to preoperative information would make a machine-learning model perform better than logistic regression at predicting which patients would experience postoperative medical complications.

## Methods

### Study design and population

This study on preoperative prediction is done in accordance with the Transparent reporting of multivariable prediction model for individual prognosis or diagnosis (TRIPOD) statement [22] and the Clinical AI Research (CAIR) checklist proposal [23]. The study is based on the Centre for Fast-track Hip and Knee Replacement database which is a prospective database on preoperative patient characteristics and enrolling consecutive patients from 7 departments between 2010 and 2017. Only cases with surgery between 2014 and 2017 were used in the present study to ensure the most up-to date data. The database is registered on ClinicalTrials.gov as a study registry (NCT01515670). Patients completed a preoperative questionnaire with nurse assistance if needed. Additional information on reimbursed prescriptions 6 months prior to surgery was acquired using the Danish National Database of Reimbursed Prescriptions (DNDRP) which records all dispensed prescriptions with reimbursement in Denmark [21]. Finally, data were combined with the Danish National Patient Registry (DNPR) for information on length of stay (counted as postoperative nights spent in hospital), 90-days readmissions with overnight stay and mortality. In case of length of stay > 4 days or readmission, patient discharge summaries were reviewed for information on postoperative morbidity and in case of insufficient information, the entire medical records were reviewed. Readmissions were only included if considered related to the surgical procedure, thus excluding planned procedures like cancer workouts, cataract surgery, etc. Readmissions due to urinary tract infection or dizziness after day 30 were also considered unrelated to the surgical procedure. In case of postoperative mortality, the entire medical record including potential readmissions, was reviewed to identify cause of death. Evaluation of discharge and medical records was performed by PP supervised by CJ. In case of disagreement, records were conferred with HK. Subsequently, causes of length of stay > 4, readmissions or mortality were classified as “medical” when related to perioperative care (renal failure, falls, pain, thrombosis, anemia, venous thromboembolism or infection etc.) and “surgical” if related to surgical technique (prosthetic infection, revision surgery, periprosthetic fracture, hip dislocation, etc.) [1]. In case of a length of stay 4–6 days with a standard discharge summary describing a successful postoperative course, it was assumed that no clinically relevant postoperative complications had occurred. If length of stay was > 6 days but with standard discharge summary, the entire medical record was evaluated to confirm that no relevant complications had occurred.

### Perioperative management

All patients had elective unilateral total hip and knee replacement in dedicated arthroplasty departments with similar fast-track protocols, including multimodal opioid sparing analgesia with high-dose (125 mg) methylprednisolone, preference for spinal anesthesia, only in-hospital thromboprophylaxis when length of stay ≤ 5 days, early mobilization, functional discharge criteria and discharge to own home [1]. There are no selection criteria for the fast-track protocol as it is considered standard of care, but we excluded patients with previous major hip or knee surgery within 90-days of THA or TKA and THA due to severe congenital joint disorder or cancer (Additional file 1).

#### Outcomes

### Primary outcome

The primary outcome was to develop a machine-learning model to predict the occurrence of “medical” complications resulting in a length of stay > 4 days or readmission and compare model performance with a traditional logistic regression model. We also investigated the performance of parsimonious models including only the top ten variables from the full machine-learning and logistic regression model, respectively.

### Secondary outcome

Secondarily we investigated the performance of the full and parsimonious machine-learning and logistic regression models when including cases with a length of stay > 4 days but no reported “medical” complications.

### Statistical analysis

Data consisted of 33 input variables, of which 7 were continuous. All variables were collected prospectively, either through the patient completed questionnaire, through the DNDRP or a combination of both (Table 1). Initially we trimmed the dataset by removing 156 patients (1.7%) who were outliers with regards to weight (< 30 kg or > 250 kg) and height (< 100 cm or > 210 cm) or where these data were missing. To reduce the risk of overfitting and allow for unbiased evaluation of model performance, data was subsequently split into a training set consisting of 18,013 (82.2%) procedures from 2014–2016 and a test set of 3913 (17.8%) procedures from 2017, as is standard in modelling of data with a temporal component [24]. These sample sizes are larger than the proposed minima of 3656, when assuming the model will explain 20% of the variability as suggested by Riley et al. [25]. The data analysis, including sample size calculation, was performed in Python and is available online at https://zenodo.org/record/7330268.

**Table 1:** Patient demographics with and without the primary outcome (length of stay > 4 days or readmissions due to “medical” morbidity) in the combined test and training dataset

Preoperative characteristics n (%) unless otherwise specified	training set (n:18,013)	test set (n:3913)
Mean age (SD)	69.0 (62.0–75.0)	70.0 (62.0–76.0)
Mean number of reimbursed prescriptions^a^ (SD)	2.0 (0.0–3.0)	2.0 (0.0–3.0)
Female gender	755 (64.0)	12,133 (58.2)
Hip arthroplasty	9918 (54.8)	2260 (57.8)
Mean weight in kg (SD)	80.5 (70.0–93.0)	81.0 (70.0–92.0)
Mean height in cm (SD)	170.0 (164.0–177.0)	170.0 (164.0–177.0)
Mean body mass index (SD)	27.5 (24.6–31.2)	27.5 (24.6–31.1)
Regular use of walking aid	552 (46.8)	4398 (21.5)
Missing	29 (2.5)	359 (1.7)
Living alone	5914 (32.9)	1381 (35.7)
With others	11,971 (66.5)	2469 (63.8)
Institution	116 (0.6)	21 (0.5)
Missing	12 (0.6)	42 (1.1)
Hemoglobin (SD)	8.6 (8.1–9.1)	8.6 (8.1–9.2)
Missing	291 (1.5)	55 (1.4)
> 2 units of alcohol/day	1382 (7.7)	286 (7.4)
Missing	57 (0.8)	36 (0.9)
Active smoker	130 (11.0)	2751 (13.2)
Missing	11 (0.9)	141 (0.7)
Cardiac disease	2527 (14.0)	529 (13.7)
Missing	17 (0.6)	53 (1.4)
Hypercholesterolemia	5396 (29.9%)	1133 (29.3%)
Missing	83 (0.5)	44 (1.2)
Hypertension	9030 (51.4)	1849 (49.5)
Missing	546 (3.0)	179 (4.6)
Pulmonary disease	1668 (9.2)	355 (9.2)
Missing	63 (0.4)	38 (1.0)
Previous cerebral attack	1038 (5.8)	213 (5.6)
Missing	157 (1.3)	77 (2.0)
Previous VTE	1331 (7.5)	283 (7.4)
Missing	283 (1.6)	66 (1.7)
Malignancy (undefined)	1469 (8.1)	134 (3.4)
Previous radically treated malignancy	1752 (9.7)	440 (11.2)
Missing	136 (0.8)	40 (1.0)
Chronic kidney disease	266 (1.5)	57 (1.5)
Missing	276 (1.5)	50 (1.3)
Family member with VTE	2235 (14.1)	430 (12.5)
Missing	2189 (12.6)	479 (12.2)
Regular snoring	266 (22.5)	5522 (26.5)
Uncertain about snoring	208 (17.6)	3781 (18.1)
Missing	259 (21.9)	3309 (15.9)
Not feeling rested	7272 (42.4)	9340 (44.8)
Uncertain about being rested	48 (4.1)	809 (3.9)
Missing	105 (8.9)	1230 (5.9)
Psychiatric disorder	1464 (8.4)	282 (7.6)
Missing	580 (3.2)	182 (4.7)
Characteristic based on combination of questionnaire and DNDRP		
Diabetes		
Diet treated diabetes^b^	251 (1.4)	52 (1.3)
Oral antidiabetics	1294 (7.2)	291 (7.5)
Insulin treated diabetes^c^	405 (2.2)	68 (1.8)
Missing	68 (0.4)	36 (0.9)

*SD* standard deviation, *VTE* venous thromboembolic event, *DNDRP* Danish National Database of Reimbursed Prescriptions

^a^Antirheumatica, steroids, anticoagulants, cardiac, cholesterol lowering, respiratory and psychotropic drugs

^b^Reported diabetes but no registered prescriptions

^c ^± oral antidiabetics

As reference model, we used logistic regression with missing values being handled by multiple imputations. All variables were then normalized to have zero mean and unit standard deviation by subtracting the original mean and dividing by the original standard deviation. In addition, we used boosted decision trees (LightGBM) [26] for the machine-learning models, as such methods work well with categorical data and missing values. We used cross entropy as the objective function for the machine-learning model.

The full machine-learning model was trained and hyperparameter optimized using the Optuna framework [27] with the Tree-structured Parzen Estimator algorithm [28] to efficiently sample hyperparameters and with a median stopping rule to minimize optimization time. The models were trained on the training data and then used for making predictions on the unseen test data (Additional file 1). We did not use cross-validation in order not to assume a constant performance over time. The model classification threshold was intentionally calibrated to include 20% of the total number of patients (positive predictive fraction of 20%). This number was chosen based on clinical assumption of available additional or rearranged resources in the Danish National Healthcare system. We also included results for positive predictive fractions of 25% and 30% to illustrate model performance under such circumstances. Furthermore, we trained two parsimonious models using machine-learning and logistic regression with only the 10 most important features. All mentioned models were calibrated using Platt’s method (Additional file 2) [29]. Calibrated risk score distributions can be found in Additional file 2. Finally, we constructed a model based on age alone (Age) to explore the added value of multiple variable prediction.

To investigate the importance of the included variables, we computed the SHapley Additive exPlanations (SHAP) values, which provide estimates on which variables contribute most to the risk score predictions [30, 31]. Finally, we investigated a potential relation between reimbursed prescribed cardiac drugs, anticoagulants, psychotropics and pulmonary drugs and age. For evaluating model performance, we computed the number of true positives (TP), false positives (FP), false negatives (FN), true negatives (TN), sensitivity (true positive rate = TP / (TP + FN)), precision (positive predictive value = TP / (TP + FP)). Since the data was quite imbalanced (about a 1:20 positive:negative ratio) we also computed the Matthews Correlation Coefficient (MCC) which is independent of class imbalance [32, 33]. The MCC ranges between -1 (the 100% wrong classifier), 0 (the random classifier), and 1 (the perfect classifier). Finally, we computed the area under the receiver operating characteristic curve (AUROC) and the area under the precision recall curve (AUPRC). To evaluate the statistical difference between the classifiers, we applied a Bayesian metric comparison P(sensitivity) [34], which is the probability that a model will perform better than the machine-learning model relative to the sensitivity. Thus, for two equally performing models P(sensitivity) is ≈ 50%.

## Results

**Fig. 1:**
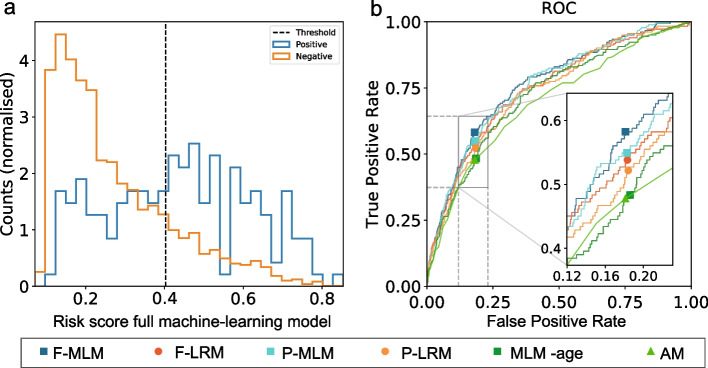
**a** Distribution of full machine learning model risk scores for patients ± the primary outcome. The dashed line marks the classification threshold of 20% positive prediction fraction. **b** Receiver operating curves (ROC) for the full machine learning model (F-MLM), full logistic regression model (F-LRM), parsimonious machine learning model (P-MLM), parsimonious logistic regression model (P-LRM) and the age-only model (AM)

Median age in the 3913 patients was 70 years (IQR 62–76), 59% were female and 58% had THA (Table 1). Details on prescribed drug types are shown in Additional File 3. Median length of stay was 2 (IQR: 1–2) days with 7.6% 90-days readmissions and the primary outcome occurring in 182 (4.7%) patients. When applying any model with a positive prediction fraction of 20% to the 3913 patients, 782 qualified as “risk-patients”. The results are summarized in Fig. 1 and Table 2.

**Table 2:** Performance of the models with a predefined positive prediction fraction of 20% for primary outcome

Positive prediction fraction 20%	TP/FP	FN/TN	Sensitivity/Precision %	MCC %	AUROC %	AUPRC %	Brier %	P (sensitivity) %
Full machine-learning model	106 / 676	76 / 3055	58.2 / 13.6	21.1	76.3	15.5	4.19	-
Full logistic regression model	97 / 685	85 / 3046	53.3 / 12.4	18.4	74.7	15.6	4.32	17.2
Parsimonious machine-learning model	100 / 682	82 / 3049	54.9 / 12.8	19.3	75.9	17.3	4.34	26.4
Parsimonious logistic regression model	90 / 692	92 / 3039	49.5 / 11.5	16.3	73.8	15.8	4.33	4.86
Age-only model	87 / 676	95 / 3055	47.8 / 11.4	15.8	69.7	12.1	38.8	3.55

*TP* true positives, *FP* false positives, *FN* false negatives, *TN* true negatives, *MCC* Matthews correlation coefficient, *AUROC* area under the operating receiver curve, *AUPRC* area under the precision recall curve P(sensitivity): probability that a model performs better than the full machine-learning model relative to sensitivity

When considering risk scores from the full machine-learning (Fig. 1a) and full logistic regression model leading to this risk-patient selection, 106 and 97 had the primary outcome, respectively. Correspondingly, the sensitivity and precision were 58.2% and 13.6% for the full machine-learning and 53.3% and 12.4% for the full logistic regression model, respectively. The full machine-learning model was superior (Fig. 1b) on all parameters (except AUPRC) compared to any of the other models, although the differences were minor (Table 2). Thus, the likelihood that the full logistic regression model or the parsimonious ML model would actually be better than the full ML model were 17.2 and 26.4% respectively. In contrast, the likelihood that the parsimonious logistic regression and the age-only model would be better that the full ML model were less than 5% (Table 2). The results were similar when using positive prediction fractions of 25% and 30%, but with the sensitivity for the full machine-learning model increasing to 64.3% and 69.2% and precision decreasing to 12.0% and 10.7%, respectively (Additional file 4). Despite age being the single most important variable, age alone had a significantly lower sensitivity at 47.8%.

**Fig. 2:**
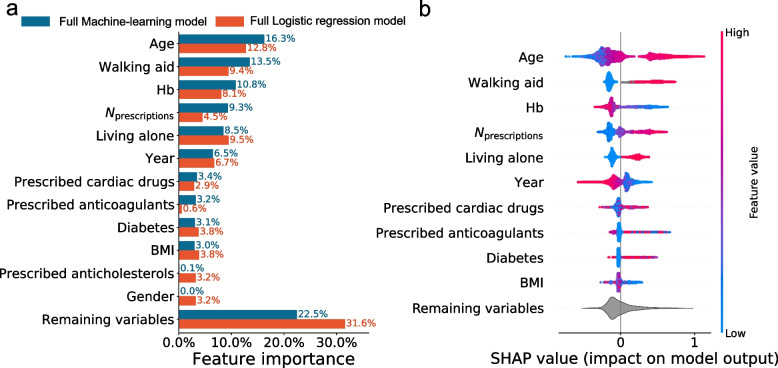
**a** The overall importance of the 10 most important variables measured by the SHAP-values for the full machine-learning and full logistic regression models on the primary outcome (LOS > 4 days or readmission due to “medical” morbidity). Only the importance of prescribed anticholesterols and gender differ between the models. The contributions of the remaining variables are summed in the bottom bar. **b** The SHAP-values for the full machine-learning model on the primary outcome, where positive values increase and negative values decrease the risk score. Each dot represents a patient and the color is related to the value of the variable with blue being lowest and red highest

When evaluating feature importance, we found a strong correlation between the full machine-learning and full logistic regression model, with age and use of walking aids being the most important variables in both (Fig. 2a). From the combined importance of variables outside the top ten, the machine-learning approach extracted more information with fewer variables than logistic regression (Fig. 1b).

**Fig. 3:**
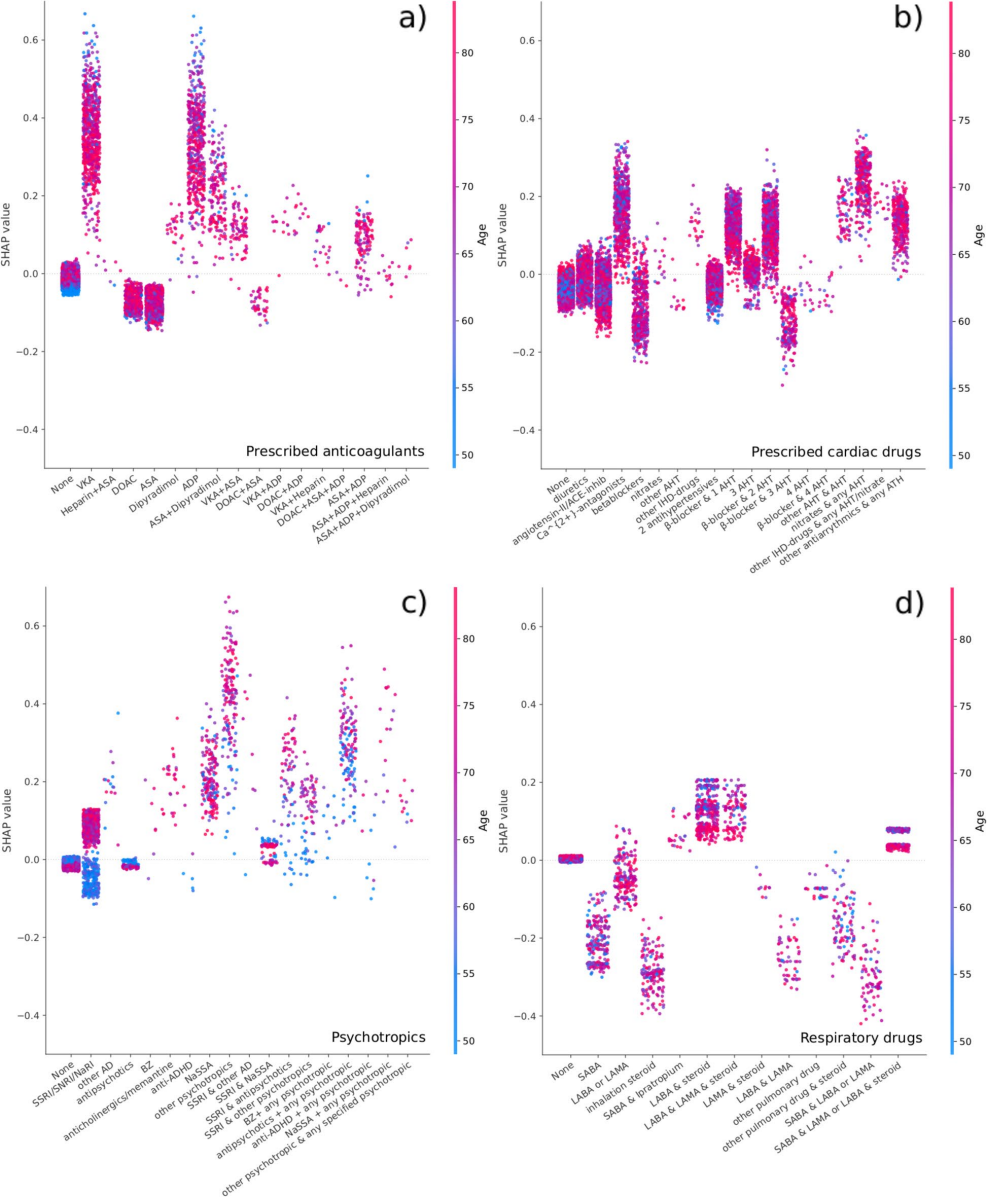
SHAP scatter-plot on the contributions to the full machine-learning model on the primary outcome (LOS > 4 days or readmission due to “medical” morbidity), for individual types of prescribed anticoagulants, cardiac drugs, psychotropics and respiratory drugs stratified by age. **a** Prescribed anticoagulants VKA: vitamin K antagonists ASA: acetylsalicylic acid DOAC: direct oral anticoagulant ADP: Adenosine diphosphate ACE: angiotensin converting enzyme. **b** Prescribed cardiac drugs ACE: angiotensin converting enzyme AHT: antihypertensive. Other AHT were defined as AHT different from diuretics ANG-II/ACE inhibitors or Ca^2+^antagonists. IHD: Ischemic heart disease. **c** Prescribed psychotropics SSRI: Selective serotonin inhibitor SNRI: Serotonin and norepinephrine reuptake inhibitor NaRI: Norepinephrine reuptake inhibitor NaSSA: Norepinephrine and specific serotonergic antidepressants. AD: antidepressants BZ: Benzodiazepines (likely underreported due to limited general reimbursement in Denmark). ADHD: Attention-deficit/hyperactivity disorder. **d** Prescribed respiratory drugs. SABA: Short-acting beta agonist LABA: long-acting beta agonist LAMA: Long-acting muscarinic antagonist

For the full machine-learning model, there was a clear signal that increasing age, number of reimbursed prescriptions, and presence of comorbidity, all contributed to an increased risk score. In contrast, a recent date of surgery and an increased hemoglobin level seemed to reduce the calculated risk (Fig. 2b). Individual analysis of the SHAP interaction values for types of anticoagulant prescriptions revealed that prescriptions on vitamin-K antagonists (VKA) or adenosine diphosphate (ADP) antagonists increased, while acetylic salicylic acid and direct oral anticoagulants (DOAC) reduced the risk score of the full machine-learning model, regardless of age (Fig. 3a). The SHAP analysis of prescribed cardiac drugs revealed that prescriptions on Ca^2+^-antagonists and betablockers in combination with one or two other antihypertensives increased the risk-score, as did prescriptions on nitrates, other antihypertensives and antiarrhythmics. For the remaining cardiac drugs, prescriptions either reduced or had minor influence, and with limited relation with age (Fig. 3b). Preoperative psychotropic prescriptions increased the risk-score except for antipsychotics (0.6%). For users of selective serotonin inhibitors there was a clear age-related distinction with the risk score being increased in elderly patients but decreased in those < 60 years (Fig. 3c). Finally, the risk score increased with prescriptions on inhalation steroid and β-blockers, and more accentuated in the younger patients (Fig. 3d). The results for our secondary outcome which included patients with a length of stay > 4 days, but no reported postoperative complications, were similar as for the primary outcome. In general, we found that the full machine-learning model was slightly superior to the others, although the differences were less than for the primary outcome. (Additional files 5 and 6). While the ten most important variables for the full machine-learning model remained unchanged, familiar disposition for venous thromboembolism replaced gender as one of the top ten important variables in the full logistic regression model (Additional file 7). Furthermore, the SHAP analysis on specific prescribed drugs demonstrated that the machine-learning model found no benefits from information on prescriptions on respiratory drugs, why all SHAP values were zero. In addition, the reduced risk with acetylsalicylic acid and DOAC prescriptions, as well as the influence of practically all cardiac drugs except for nitrates, other antihypertensives and antiarrhythmics, was attenuated (Additional file 8).

## Discussion

We found that using a machine-learning algorithm including all 33 available variables and a parsimonious machine-learning-algorithm encompassing only the 10 most important predictors improved prediction of patients at increased risk of having a length of stay > 4 days or readmissions due to medical complications compared to traditional logistic regression models. Thus, despite similarities in weighting of predictor variables, using the full machine-learning model resulted in approximately 5% increase in correctly identified risk-patients compared to the full logistic regression model. This corresponded to an increase in AUROC of about 1.5, which is about 3 times larger than what was found in a study investigating potential benefits of machine-learning for the NSQIP risk calculator [35].

In contrast, when also including patients having a length of stay > 4 days but without a well-defined complication as an outcome, the parsimonious machine-learning model was slightly worse than a traditional logistic regression model including all variables. Wei et al*.* used an artificial neural network model to predict same-day discharge after TKA, based on the NSQUIP database from 2018 and found that six of the ten most important variables were the same compared with logistic regression, similar to our findings [36].

However, patients with one-day length of stay were intentionally excluded due to variations in in-patient vs. out-patient registration [36]. A previous systematic review found that machine-learning algorithms may provide better prediction of postoperative outcomes in THA and TKA [37]. The authors concluded that such models performed best at predicting postoperative complications, pain and patient reported outcomes and were less accurate at predicting readmissions and reoperations [37]. That machine-learning algorithms may improve prediction of complications after THA and TKA compared to traditional logistic regression was also found by Shah et al. who used an automated machine-learning framework to predict selected major complications after THA [13]. However, theirs was a retrospective study based on diagnostic and administrative coding and the selected complications occurred only in 0.61% of patients, potentially limiting clinical relevance. In contrast, we aimed at identifying a cohort which would comprise 20% of patients in which we found about 60% of all medical complications. This we believe, is within the means of the Danish socialized healthcare system to allocate additional resources for intensified perioperative care and with both patient-related and economic benefits due to potentially avoided complications and costs. In this context, the models using 25% and 35% positive prediction thresholds demonstrated that the gain in sensitivity leading to identification of 14–24 more patients with complications was at the cost of 196 to 391 more patients being “wrongly” classified as risk patients. Age has traditionally been a major factor when predicting surgical outcomes and remained the single most important predictor in our study. However, although elderly patients had increased risk of postoperative complications, likely related to decline of physical reserves [38], the use of chronological age alone was inferior compared to both machine-learning and logistic regression models incorporating comorbidity and functional status. Thus, using age by itself for identifying the high-risk population resulted in missing 18% of the “true risk-patients” (87 compared to 106 in the full ML model).

We used the SHAP values for estimation of the impact of the included variables. The SHAP values show which variables contribute most to the risk-score, thus providing a better understanding of the otherwise “black-box” machine-learning model. This approach was also used by Bonde and colleagues, who used deep neural networks to predict postoperative complications across several different surgical procedures [10]. In our study, the SHAP analysis on unique Danish registry data on reimbursed prescriptions, unsurprisingly found a considerable increase in risk-score with an increasing number of prescriptions, especially in elderly patients However, this is a complex relationship where some patients benefit from their treatments, while other may suffer from undesirable side-effects. Nevertheless, the information from the SHAP analysis in machine-learning studies may provide inspiration for new hypothesis-generating studies on risk-factors, e.g. on the potential differences in risk-profile between having preoperative prescribed VKA and DOAKs found in our study. Also, the age-related differences in risk from SSRI’s could guide further studies on “deprescription”.

Another important requirement for machine-learning-algorithms to be clinically useful is user friendliness and not depending on excessive additional data collection by the attending clinicians [9]. In this context, it was disappointing that the parsimonious machine-learning algorithm with only the ten most important variables was slightly worse at predicting the secondary outcome than the full logistic regression model. This could be due to a length of stay > 4 days but without described medical complications more often is related to social and logistical factors not contained within the ten most important patient-related preoperative variables, e.g., having a supportive network, availability of homecare etc. Thus, the information gained by the combination of all available information may be of further importance when merely using LOS as outcomes in prediction studies. However, it also highlights the need for as much detailed, and preferably non-binary, data as possible to fulfill the true potential of machine-learning algorithms. In contrast to several other machine-learning studies, our dataset included only one paraclinical variable, which was preoperative hemoglobin. Although the inclusion of other laboratory tests such as albumin, sodium and alkaline phosphatase has been found to be of importance in some machine-learning algorithms [10, 39] they are not standard in fast-track protocols and not easy to interpret from a pathophysiological point of view. Also, most decisions on intensified postoperative care in elective surgery will likely need to be conducted preoperatively, as there is an increasing need to prioritize limited health-care resources. Thus, although postoperative information such as duration of surgery, perioperative blood length of stays or postoperative hemoglobin have been included in other studies [39], we decided against the use of peri- and postoperative data. The same approach has been used by Ramkumar et al*.* who used U.S. National Inpatient Sample data including 15 preoperative variables, to predict length of stay, patient charges and disposition after both TKA and THA [17, 40]. However, these studies were not conducted in a socialized health care system, and their main focus was on the need for differentiated payment bundles and without specific information on the reason for increased length of stay or non-home discharge [40].

Our study has some other limitations. First, one of the strengths of machine learning compared to logistic regression is the analysis of multilevel continuous data, whereas we included only a limited number of, often binary, preoperative variables. This could have limited the full realization of our machine-learning model. As previously mentioned, we excluded intraoperative information, including type of anesthesia, surgical approach etc. all of which may influence postoperative outcomes. The observational design of this study means that we cannot exclude unmeasured confounding or confounding by indication. Also, despite that the DNDRP has a near complete registration of dispensed medicine in Denmark, some types or drugs, especially benzodiazepines, are exempt from general reimbursement and thus not sufficiently captured [21]. Furthermore, it is doubtful whether the patients used all types of drugs at the time of surgery (e.g. heparin which is rarely for long-term use). The classification of a complication being “medical” depended on review of the discharge records could also introduce bias. However, we believe our approach to be superior to depending only on diagnostic codes which often are inaccurate [41] and provide limited details on whether the complication may be attributed to a medical or surgical adverse event. The strengths of our study include the use of national registries with high degree of completion (> 99% of all somatic admissions in case of the DNDRP) [42], prospective recording of comorbidity, extensive information on prescription patterns 6 months prior to surgery. Finally, the similar established enhanced recovery protocols in all departments assured that all patients were treated according to the most modern evidence-based principles. Thus, our analysis is based on well-defined time-relevant clinical treatments.

In summary, our results suggest that machine-learning-algorithms may provide slight, but clinically relevant, improved predictions for defining patients in high-risk of medical complications after fast-track THA and TKA compared to logistic regression models. Future studies could benefit from using such algorithms to find a manageable population of patients who may benefit the most from intensified perioperative care.

### Supplementary Information

**Additional file 1.** Flowchart of the study population and final sample size.

** Additional file 2.** Calibration plots of the machine-learning and logistic regression models.

** Additional file 3.** Details on specific drugs with reimbursed prescriptions 6 months preoperatively.

** Additional file 4.** Performance of the different models with a predefined positive prediction fraction of 25 and 30 for the primary outcome (LOS >4 days or readmission due to “medical” morbidity.

** Additional file 5.** Performance of different models for the secondary outcome (LOS >4 days or readmissions due to “medical” morbidity or LOS >4 days but without recorded morbidity).

** Additional file 6.** 1a) Distribution of full machine-learning model risk-scores for patients +/- the secondary outcome 1b) Receiver operating curves.

** Additional file 7.** 2a) The overall importance of the 10 most important variables measured by the SHAP-values for the full machine-learning and full logistic regression models for the secondary outcome 2b) The SHAP-values for the full machine-learning model.

** Additional file 8.** SHAP scatter-plot on the contributions to the full machine-learning model on outcome B for individual types of prescribed anticoagulants, cardiac drugs, psychotropics and respiratory drugs stratified by age.

## Data Availability

The original dataset is not publicly available due to Danish data-protection law but can be acquired from the corresponding author by request. All statistical code can be freely accessed from https://zenodo.org/record/7330268.

## Abbreviations

THA: Total hip arthroplasty
TKA: Total knee arthroplasty
TRIPOD: Transparent reporting of multivariable prediction model for individual prognosis or diagnosis
CAIR: Clinical AI Research
DNDRP: Danish National Database of Reimbursed Prescriptions
DNPR: Danish National Patient Registry
SHAP: SHapley Additive exPlanations
TP: True positives
FP: False positives
FN: False negatives
TN: True negatives
MCC: Matthews Correlation Coefficient
AUROC: Area under the receiver operating characteristic curve
AUPRC: Area under the precision recall curve
VKA: Vitamin K antagonist
DOAC: Dual oral anticoagulants
SSRI: Selective serotonin reuptake inhibitors
