# Supplementary material for: Machine-learning vs. logistic regression for preoperative prediction of medical morbidity after fast-track hip and knee arthroplasty—a comparative study
Source: BMC Anesthesiol. 2023 Nov 29;23:391. doi: 10.1186/s12871-023-02354-z (PMC10685559; doi:10.1186/s12871-023-02354-z)
Supplement: Supplementary file 1 — Additional file 1. Flowchart of the study population and final sample size. [file 12871_2023_2354_MOESM1_ESM.pdf]

Additional file 1

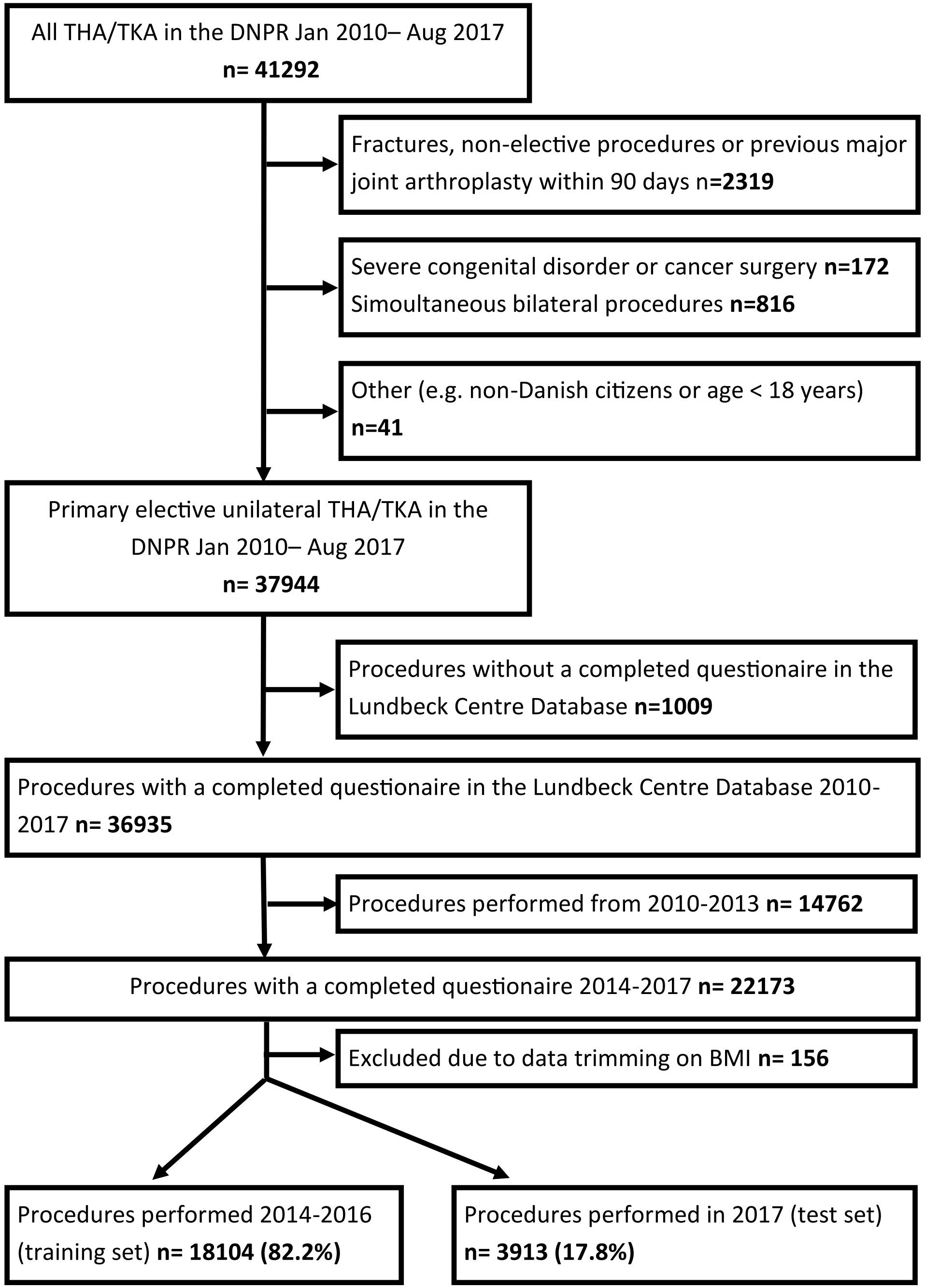

Flowchart of the study population and final sample size. THA: total hip arthroplasty TKA: total knee arthroplasty DNPR: the Danish National Patient Registry
