# Supplementary material for: Machine-learning vs. logistic regression for preoperative prediction of medical morbidity after fast-track hip and knee arthroplasty—a comparative study
Source: BMC Anesthesiol. 2023 Nov 29;23:391. doi: 10.1186/s12871-023-02354-z (PMC10685559; doi:10.1186/s12871-023-02354-z)
Supplement: Supplementary file 2 — Additional file 2. Calibration plots of the machine-learning and logistic regression models. [file 12871_2023_2354_MOESM2_ESM.pdf]

Additional file 2

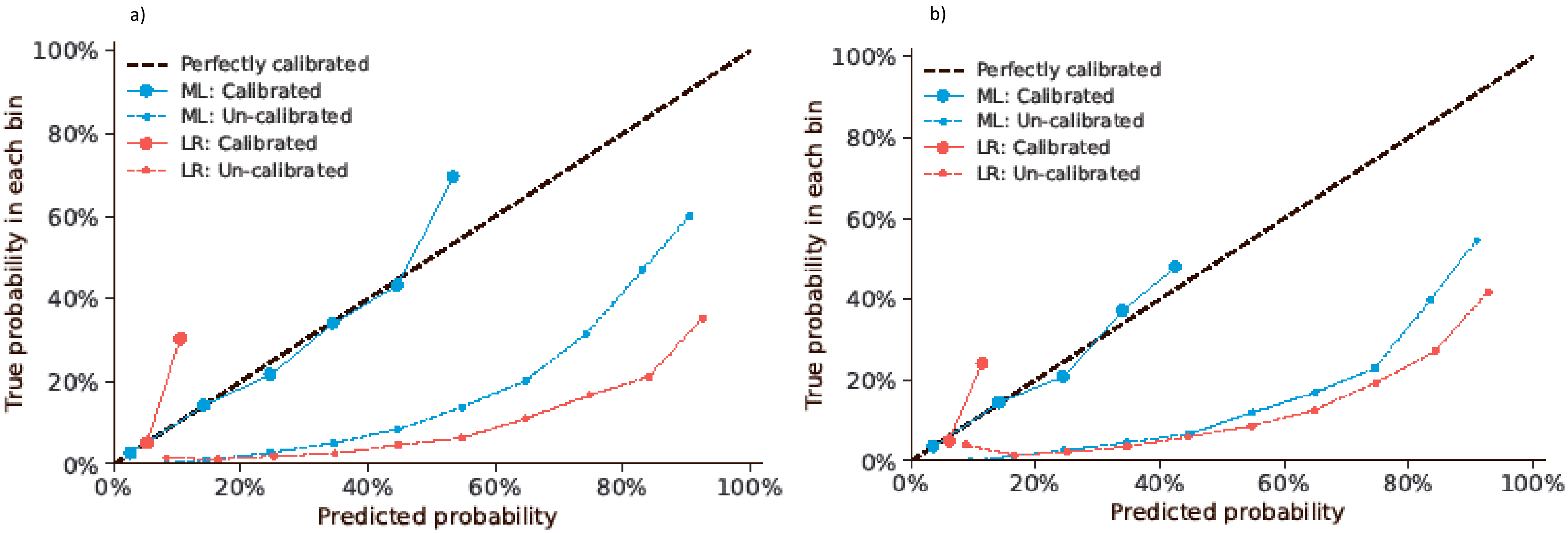

Calibration plot of the machine learning model (ML) and the logistic regression (LR) for both the calibrated (a) and un-calibrated (b) models for the primary outcome.
