# Supplementary material for: Machine-learning vs. logistic regression for preoperative prediction of medical morbidity after fast-track hip and knee arthroplasty—a comparative study
Source: BMC Anesthesiol. 2023 Nov 29;23:391. doi: 10.1186/s12871-023-02354-z (PMC10685559; doi:10.1186/s12871-023-02354-z)
Supplement: Supplementary file 3 — Additional file 3. Details on specific drugs with reimbursed prescriptions 6 months preoperatively. [file 12871_2023_2354_MOESM3_ESM.pdf]

Additional file 3

Details on specific drugs with reimbursed prescriptions 6 months preoperatively.
Numbers are n (%)

Reimbursed prescriptions within 3 months preoperatively	training set (n:18104)	test set (n:3913)
Anticoagulants		
none	13570 (75.0)	2953 (75.5)
VKA	729 (4.0)	127 (3.2)
Heparin & Acetylsalicylic acid	6 (0.0)	1 (0.0)
DOAC	526 (2.9)	181 (4.6)
Acetylsalicylic acid	2235 (12.3)	462 (11.8)
Dipyradimol	31 (0.2)	3 (0.1)
ADP-antagonist	522 (2.9)	122 (3.1)
Acetylsalicylic acid & Dipyradimol	169 (0.9)	16 (0.4)
VKA & Acetylsalicylic acid	81 (0.4)	7 (0.2)
DOAC & Acetylsalicylic acid	42 (0.2)	5 (0.1)
VKA & ADP-antagonist	13 (0.1)	2 (0.1)
DOAC & ADP-antagonist	12 (0.1)	5 (0.1)
VKA & Heparin	22 (0.1)	0 (0.0)
DOAC & Acetylsalicylic acid & ADP-antagonist	3 (0.0)	1 (0.0)
Acetylsalicylic acid & ADP-antagonist	124 (0.7)	26 (0.7)
Acetylsalicylic acid & ADP-antagonist & Heparin	12 (0.1)	1 (0.0)
Acetylsalicylic acid & ADP-antagonist & Dipyradimol	7 (0.0)	1 (0.0)
Cardiac prescriptions		
none	7741 (42.8)	1780 (45.5)
diuretics	1070 (5.9)	191 (4.9)
angiotensin-II/ACE-inhibitors	2287 (12.6)	528 (13.5)
Ca^{2+} antagonists	688 (3.8)	140 (3.6)
β-blocker	492 (2.7)	96 (2.5)
nitrates	14 (0.1)	5 (0.1)
other antihypertensives	12 (0.1)	0 (0.0)
other types of medication for IHD	22 (0.1)	1 (0.0)
2 antihypertensives	2360 (13.0)	513 (13.1)
β-blocker & 1 antihypertensive[1]	966 (5.3)	195 (5.0)
3 antihypertensives	515 (2.8)	83 (2.1)
β-blocker & 2 antihypertensives[1]	902 (5.0)	168 (4.3)
β-blocker & 3 antihypertensives[1]	235 (1.3)	55 (1.4)
4 antihypertensives	16 (0.1)	4 (0.1)
β-blocker & 4 antihypertensives	15 (0.1)	6 (0.2)
other antihypertensive & antihypertensives[1]	78 (0.4)	18 (0.5)
nitrates & any hypertensive	323 (1.8)	57 (1.5)
other drugs for IHD & any antihypertensive and/or nitrate	16 (0.1)	4 (0.1)
other antiarrhythmics & any antihypertensives	352 (1.9)	69 (1.8)
Anticholesterols		
none	12665 (70.0)	2762 (70.6)
statins	5218 (28.8)	1105 (28.2)
other anti-lipids	118 (0.7)	24 (0.6)
statins +other anti-lipids	103 (0.6)	22 (0.6)
Systemic steroids	1038 (5.7)	234 (6.0)

Antirheumatics		
none	17709 (97.8)	3822 (97.7)
disease-modifying antirheumatic drugs	392 (2.2)	91 (2.3)
other antirheumatics	3 (0.0)	0 (0.0)
Respiratory prescriptions		
none	16256 (89.8)	3498 (89.4)
SABA	235 (1.3)	54 (1.4)
LABA or LAMA	194 (1.1)	42 (1.1)
inhalation steroid only	176 (1.0)	43 (1.1)
SABA & Ipratropium (+/- others)	24 (0.1)	0 (0.0)
LABA & steroid	432 (2.4)	87 (2.2)
LABA & LAMA & steroid	115 (0.6)	26 (0.7)
LAMA & steroid	10 (0.1)	1 (0.0)
LABA & LAMA	56 (0.3)	31 (0.8)
other pulmonary drugs	26 (0.1)	9 (0.2)
other pulmonary drugs & steroid	95 (0.5)	12 (0.3)
SABA & LABA or LAMA	76 (0.4)	26 (0.7)
SABA & LABA or LAMA & steroid	409 (2.3)	84 (2.1)
Psychotropic prescriptions		
none	16113 (89.0)	3496 (89.3)
SSRI/SNRI/NaRI	1055 (5.8)	209 (5.3)
other antidepressants	16 (0.1)	2 (0.1)
antipsychotics	104 (0.6)	20 (0.5)
benzodiazepines[2]	7 (0.0)	0 (0.0)
anti-cholinergics or memantine	27 (0.1)	6 (0.2)
anti-ADHD drugs	7 (0.0)	4 (0.1)
NaSSA	177 (1.0)	32 (0.8)
other psychotropics	166 (0.9)	44 (1.1)
SSRI + other antidepressants	9 (0.0)	1 (0.0)
SSRI + NaSSA	86 (0.5)	16 (0.4)
SRRI + antipsychotics	80 (0.4)	18 (0.5)
SRRI + other psychotropics	72 (0.4)	19 (0.5)
benzodiazepines + any psychotropic	11 (0.1)	4 (0.1)
antipsychotics + any psychotropic	137 (0.8)	32 (0.8)
anti-ADHD + any psychotropic	11 (0.1)	3 (0.1)
NaSSA + any psychotropic	16 (0.1)	6 (0.2)
other psychotropics + any specified psychotropic	10 (0.1)	1 (0.0)

VKA: vitamin K antagonists DOAC: direct oral anticoagulant ADP: Adenosine diphosphate ACE: angiotensin converting enzyme IHD: Ischemic heart disease SABA: Short-acting beta agonist LABA: long-acting beta agonist LAMA: Long-acting muscarinic antagonist SSRI: Selective serotonin inhibitor SNRI: Serotonin and norepinephrine reuptake inhibitor NaRI: Norepinephrine reuptake inhibitor NaSSA: Norepinephrine and specific serotonergic antidepressants

[1]either diuretics, ACE/ANG-II inhibitors or Ca^{2+}antagonists [2] likely underreported due to limited general reimbursement for benzodiazepines in Denmark
