# Supplementary material for: Machine-learning vs. logistic regression for preoperative prediction of medical morbidity after fast-track hip and knee arthroplasty—a comparative study
Source: BMC Anesthesiol. 2023 Nov 29;23:391. doi: 10.1186/s12871-023-02354-z (PMC10685559; doi:10.1186/s12871-023-02354-z)
Supplement: Supplementary file 4 — Additional file 4. Performance of the different models with a predefined positive prediction fraction of 25 and 30 for the primary outcome (LOS >4 days or readmission due to “medical” morbidity. [file 12871_2023_2354_MOESM4_ESM.pdf]

Additional file 4

Performance of the different models with a predefined positive prediction fraction of 25 and 30 for the primary outcome (LOS >4 days or readmission due to "medical" morbidity.

**Positive prediction fraction 25%**	TP	FP	FN	TN	Sensitivity %	Precision %	MCC %	AUROC %	AUPRC %	Brier %	P(sensitivity) %
Full machine-learning model	120	858	62	2873	65.9	12.3	20.9	77.0	15.3	4.32	-
Full logistic regression model	108	870	74	2861	59.3	11.0	17.5	74.6	15.6	4.32	10.4
Parsimonious machine-learning model	114	864	68	2867	62.6	11.7	19.2	74.9	14.1	4.35	26.2
Parsimonious logistic regression model	103	875	79	2856	56.6	10.5	16.1	73.6	15.2	4.33	3.9
Age-model	94	824	88	2907	51.6	10.2	14.7	69.7	12.2	38.8	1.2
**Positive prediction fraction 30%**	TP	FP	FN	TN	Sensitivity %	Precision %	MCC %	AUROC %	AUPRC %	Brier %	P(sensitivity) %
Full machine-learning model	130	1043	52	2688	71.4	11.1	20.0	77.0	15.3	4.32	-
Full logistic regression model	117	1056	65	2675	64.2	10.0	16.5	74.6	15.6	4.32	8.4
Parsimonious machine-learning model	124	1049	58	2682	68.1	10.6	18.4	74.9	14.1	4.35	30.0
Parsimonious logistic regression model	118	1055	64	2676	64.8	10.1	16.8	73.6	15.2	4.33	14.0
Age-model	100	955	82	2776	54.9	9.5	13.9	69.7	12.2	38.8	0.9

TP: true positives FP: false positives FN: false negatives TN: true negatives MCC: Matthews correlation coefficient AUROC: area under the receiver operating curve AUPRC: area under the precision recall curve P(sensitivity): probability that the model performs better than the machine-learning model relative to sensitivity.
