# Supplementary material for: Machine-learning vs. logistic regression for preoperative prediction of medical morbidity after fast-track hip and knee arthroplasty—a comparative study
Source: BMC Anesthesiol. 2023 Nov 29;23:391. doi: 10.1186/s12871-023-02354-z (PMC10685559; doi:10.1186/s12871-023-02354-z)
Supplement: Supplementary file 5 — Additional file 5. Performance of different models for the secondary outcome (LOS >4 days or readmissions due to “medical” morbidity or LOS >4 days but without recorded morbidity). [file 12871_2023_2354_MOESM5_ESM.pdf]

Additional File 5

Performance of different models for the secondary outcome (LOS >4 days or readmissions due to “medical” morbidity or LOS >4 days but without recorded morbidity)

**Positive prediction fraction 20%**	TP	FP	FN	TN	Sensitivity %	Precision %	MCC %	AUROC %	AUPRC %	Brier %	P(sensitivity) %
Full machine-learning model	117	665	112	3019	51.1	15.0	19.4	75.0	18.1	5.23	-
Full logistic regression model	115	667	114	3017	50.2	14.7	18.9	74.1	16.7	5.35	46.4
Parsimonious machine-learning model	109	673	120	3011	47.6	13.9	17.2	72.1	15.8	5.33	35.2
Parsimonious logistic regression model	109	673	120	3011	47.6	13.9	17.2	72.9	16.7	5.37	22.6
Age-model	102	661	127	3023	44.5	13.4	15.8	68.7	13.4	38.3	10.3
**Positive prediction fraction 25%**	TP	FP	FN	TN	Sensitivity %	Precision %	MCC %	AUROC %	AUPRC %	Brier %	P(sensitivity) %
Full machine-learning model	128	850	101	2834	55.9	13.1	17.8	75.0	18.1	5.23	-
Full logistic regression model	133	845	96	2839	58.1	13.6	19.1	74.1	16.7	5.35	68.0
Parsimonious machine-learning model	121	857	108	2827	52.8	12.3	16.3	72.1	15.8	5.33	25.5
Parsimonious logistic regression model	127	851	102	2833	55.5	13.0	17.5	72.9	16.7	5.37	46.6
Age-model	113	805	116	2879	49.3	12.3	15.2	68.7	13.4	38.3	17.2
**Positive prediction fraction 30%**	TP	FP	FN	TN	Sensitivity %	Precision %	MCC %	AUROC %	AUPRC %	Brier %	P(sensitivity) %
Full machine-learning model	146	1027	83	2657	63.4	12.4	18.4	75.0	18.1	5.23	-
Full logistic regression model	144	1029	85	2655	62.9	12.3	17.9	74.1	16.7	5.35	42.4
Parsimonious machine-learning model	135	1038	94	2651	59.0	11.5	15.8	72.1	15.8	5.33	14.9
Parsimonious logistic regression model	140	1033	89	2651	61.1	11.9	17.0	72.9	16.7	5.37	28.3
Age-model	122	933	107	2751	53.3	11.6	14.8	68.7	13.4	38.3	7.9

TP: true positives FP: false positives FN: false negatives TN: true negatives MCC: Matthews correlation coefficient AUROC: area under the receiver operating curve AUPRC: area under the precision recall curve P(sensitivity): probability that a model performs better than the machine-learning model relative to sensitivity.
