# Supplementary material for: Machine-learning vs. logistic regression for preoperative prediction of medical morbidity after fast-track hip and knee arthroplasty—a comparative study
Source: BMC Anesthesiol. 2023 Nov 29;23:391. doi: 10.1186/s12871-023-02354-z (PMC10685559; doi:10.1186/s12871-023-02354-z)
Supplement: Supplementary file 6 — Additional file 6. 1a) Distribution of full machine-learning model risk-scores for patients +/- the secondary outcome 1b) Receiver operating curves. [file 12871_2023_2354_MOESM6_ESM.pdf]

Additional file 6 Figure 1a-b

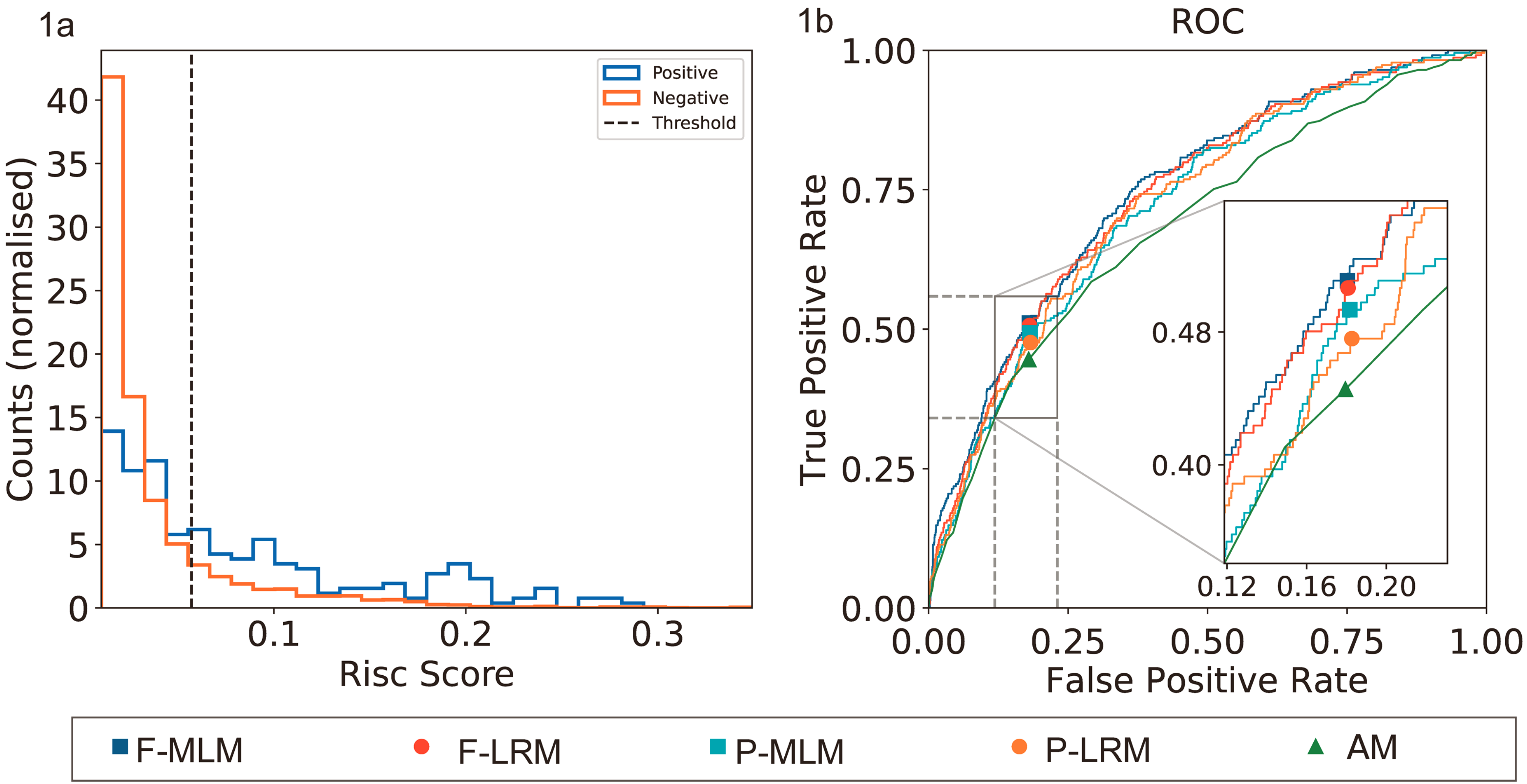

1a) Distribution of full machine-learning model risk-scores for patients +/- the secondary outcome (LOS >4 days or readmissions due to "medical" morbidity or LOS >4 days with no recorded morbidity). The dashed line marks the classification threshold of a 20% positive prediction fraction.

1b) Receiver operating curves (ROC) for the full machine-learning model (F-MLM), full logistic regression model (F-LRM), parsimonious machine-learning model (P-MLM), parsimonious logistic regression model (P-LRM) and the age-only model (AM).
