# Supplementary material for: Machine-learning vs. logistic regression for preoperative prediction of medical morbidity after fast-track hip and knee arthroplasty—a comparative study
Source: BMC Anesthesiol. 2023 Nov 29;23:391. doi: 10.1186/s12871-023-02354-z (PMC10685559; doi:10.1186/s12871-023-02354-z)
Supplement: Supplementary file 7 — Additional file 7. 2a) The overall importance of the 10 most important variables measured by the SHAP-values for the full machine-learning and full logistic regression models for the secondary outcome 2b) The SHAP-values for the full machine-learning model. [file 12871_2023_2354_MOESM7_ESM.pdf]

Additional file 7

Figure 2a-b

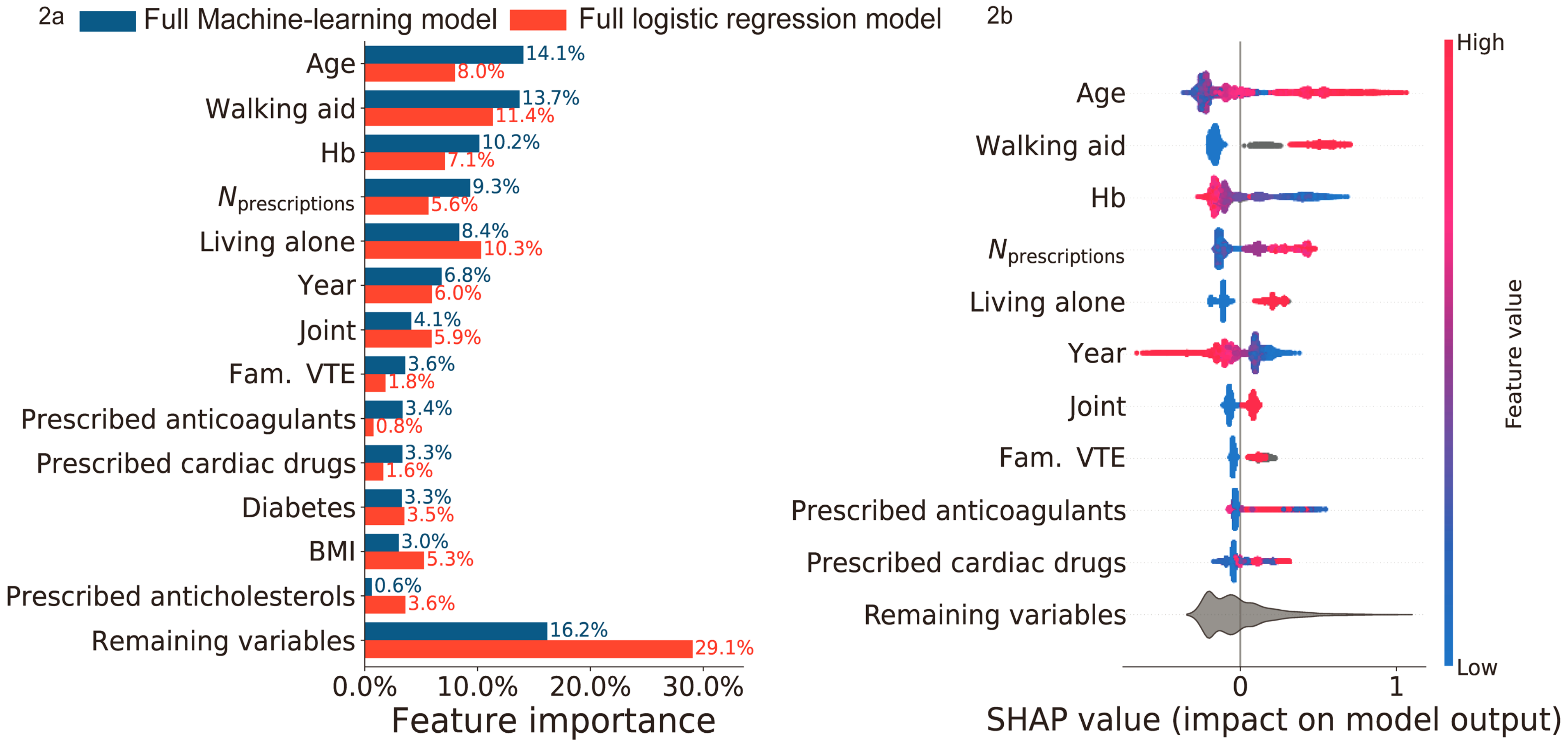

2a) The overall importance of the 10 most important variables measured by the SHAP-values for the full machine-learning and full logistic regression models for the secondary outcome (LOS >4 days or readmissions due to “medical” morbidity or LOS >4 days with no recorded morbidity).
Only the importance of prescribed anti-cholesterols and familiar disposition for venous thromboembolism differed between the models. The contributions of the remaining variables are summed in the bottom bar.
2b) The SHAP-values for the full machine-learning model. Positive SHAP-values increase the risk score while negative values decrease the risk score. Each dot repre-
