# Supplementary material for: Machine-learning vs. logistic regression for preoperative prediction of medical morbidity after fast-track hip and knee arthroplasty—a comparative study
Source: BMC Anesthesiol. 2023 Nov 29;23:391. doi: 10.1186/s12871-023-02354-z (PMC10685559; doi:10.1186/s12871-023-02354-z)
Supplement: Supplementary file 8 — Additional file 8. SHAP scatter-plot on the contributions to the full machine-learning model on outcome B for individual types of prescribed anticoagulants, cardiac drugs, psychotropics and respiratory drugs stratified by age. [file 12871_2023_2354_MOESM8_ESM.pdf]

Additional file 8
SHAP scatter-plot on the contributions to the full machine-learning model on outcome B (LOS >4 days or readmission due to “medical” morbidity), for individual types of prescribed anticoagulants, cardiac drugs, psychotropics and respiratory drugs stratified by age

Legend:
3a) Prescribed anticoagulants
VKA: vitamin K antagonists ASA: acetylsalicylic acid DOAC: direct oral anticoagulant ADP: Adenosine diphosphate ACE: angiotensin converting enzyme
3b) Prescribed cardiac drugs
ACE: angiotensin converting enzyme AHT: antihypertensive. Other AHT were defined as AHT different from diuretics ANG-II/ACE inhibitors or Ca2+antagonists. IHD: Ischemic heart disease
3c) Prescribed psychotropics
SSRI: Selective serotonin inhibitor SNRI: Serotonin and norepinephrine reuptake inhibitor NaRI: Norepinephrine reuptake inhibitor NaSSA: Norepinephrine and specific serotonergic antidepressants. AD: antidepressants BZ: Benzodiazepines (likely underreported due to limited general reimbursement in Denmark). ADHD: Attentiondeficit/hyperactivity disorder
3d) Prescribed respiratory drugs
The model found no additional information from this variable why all values equal 0.
SABA: Short-acting beta agonist LABA: long-acting beta agonist LAMA: Long-acting muscarinic antagonist.

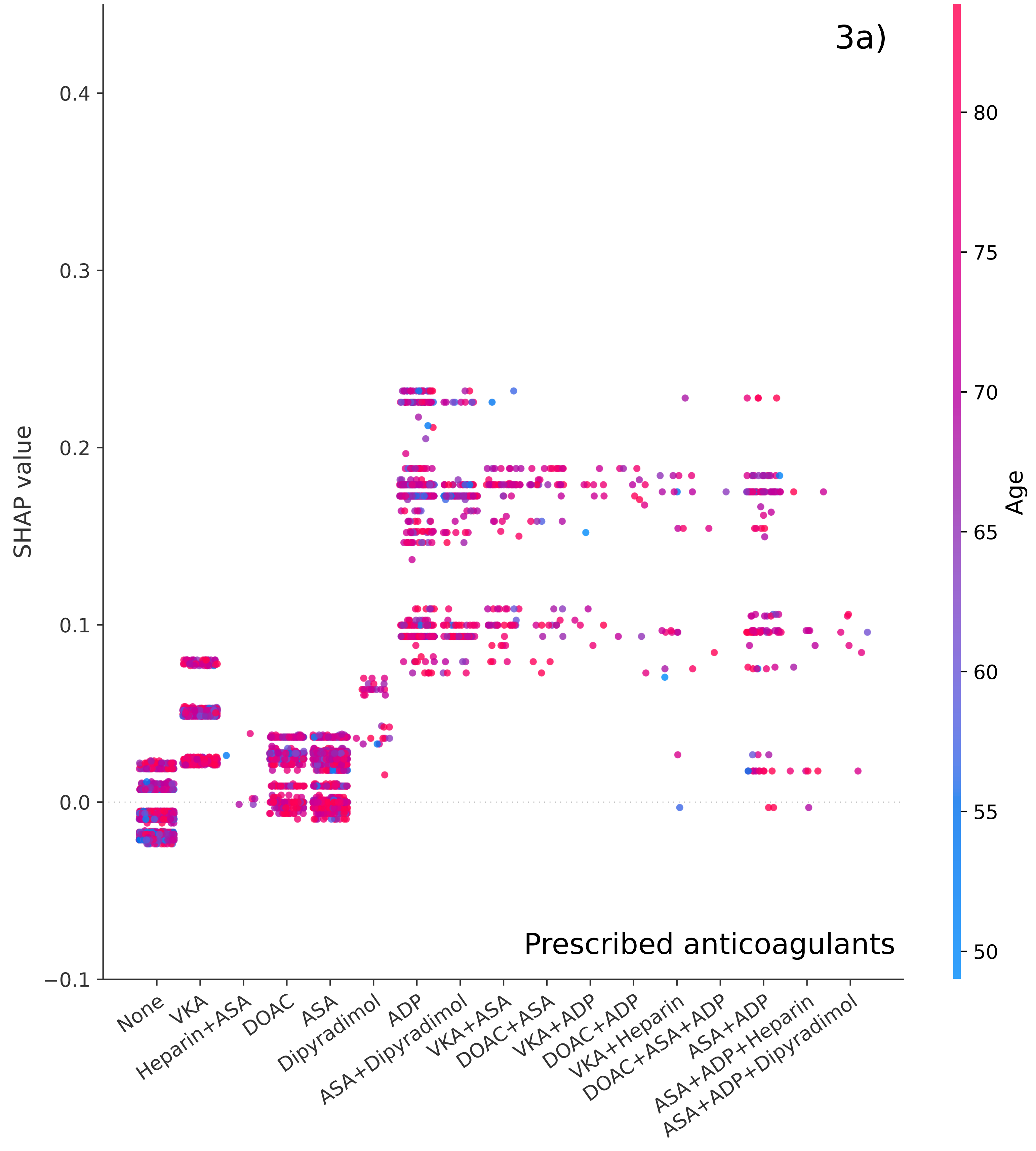
3a)
SHAP value
0.4
0.3
0.2
0.1
0.0
−0.1
Prescribed anticoagulants
None
VKA
Heparin+ASA
DOAC
ASA
Dipyradimol
ADP
ASA+Dipyradimol
VKA+ASA
DOAC+ASA
VKA+ADP
DOAC+ADP
VKA+Heparin
DOAC+ASA+ADP
ASA+ADP
ASA+ADP+Heparin
ASA+ADP+Dipyradimol
Age
80
75
70
65
60
55
50

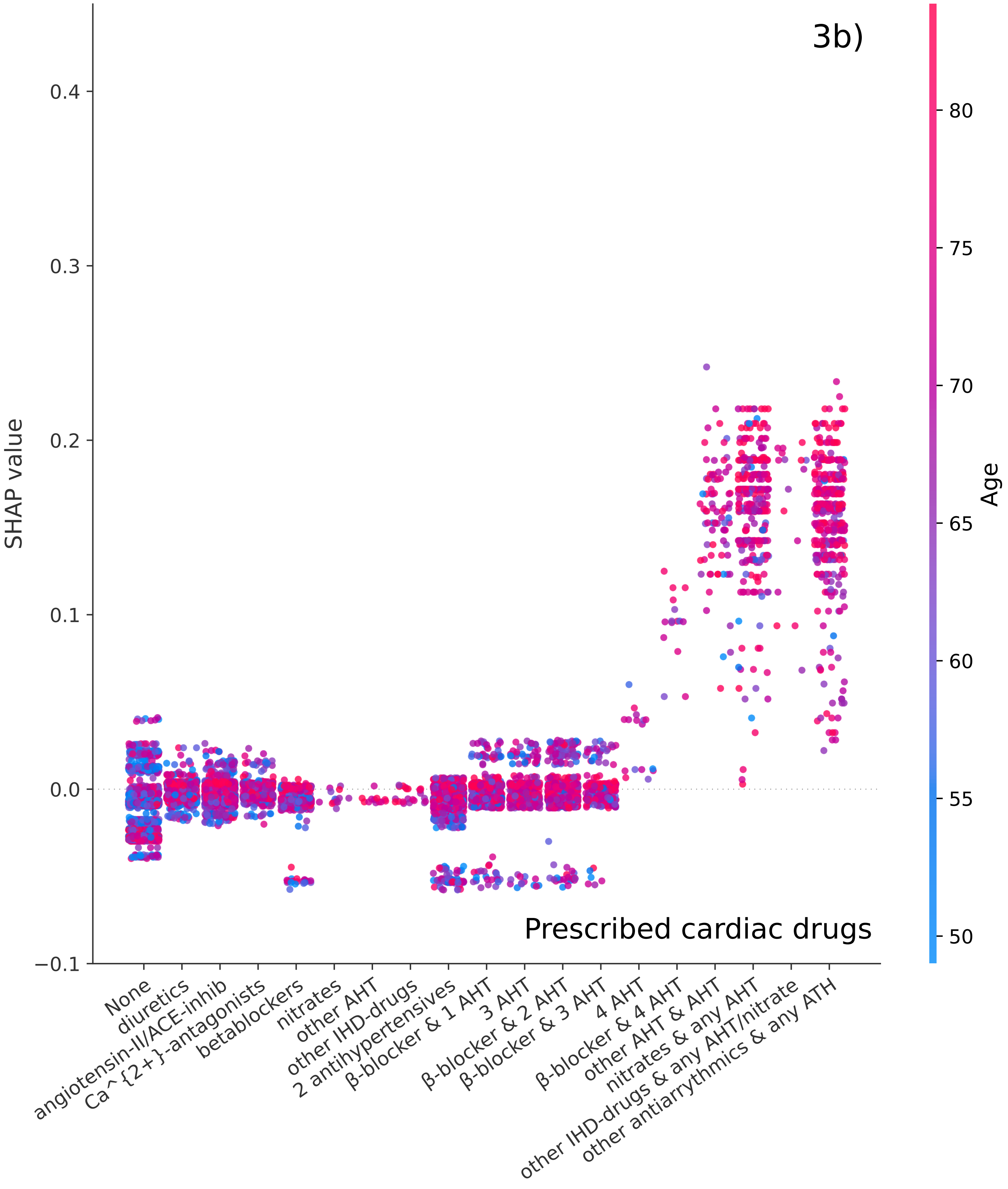
3b)
SHAP value
0.4
0.3
0.2
0.1
0.0
−0.1
Prescribed cardiac drugs
Age
80
75
70
65
60
55
50
None
diuretics
angiotensin-II/ACE-inhib
Ca^{2+}-antagonists
betablockers
nitrates
other AHT
other IHD-drugs
2 antihypertensives
β-blocker & 1 AHT
3 AHT
β-blocker & 2 AHT
β-blocker & 3 AHT
4 AHT
β-blocker & 4 AHT
other AHT & AHT
nitrates & any AHT
other IHD-drugs & any AHT/nitrate
other antiarrythmics & any ATH

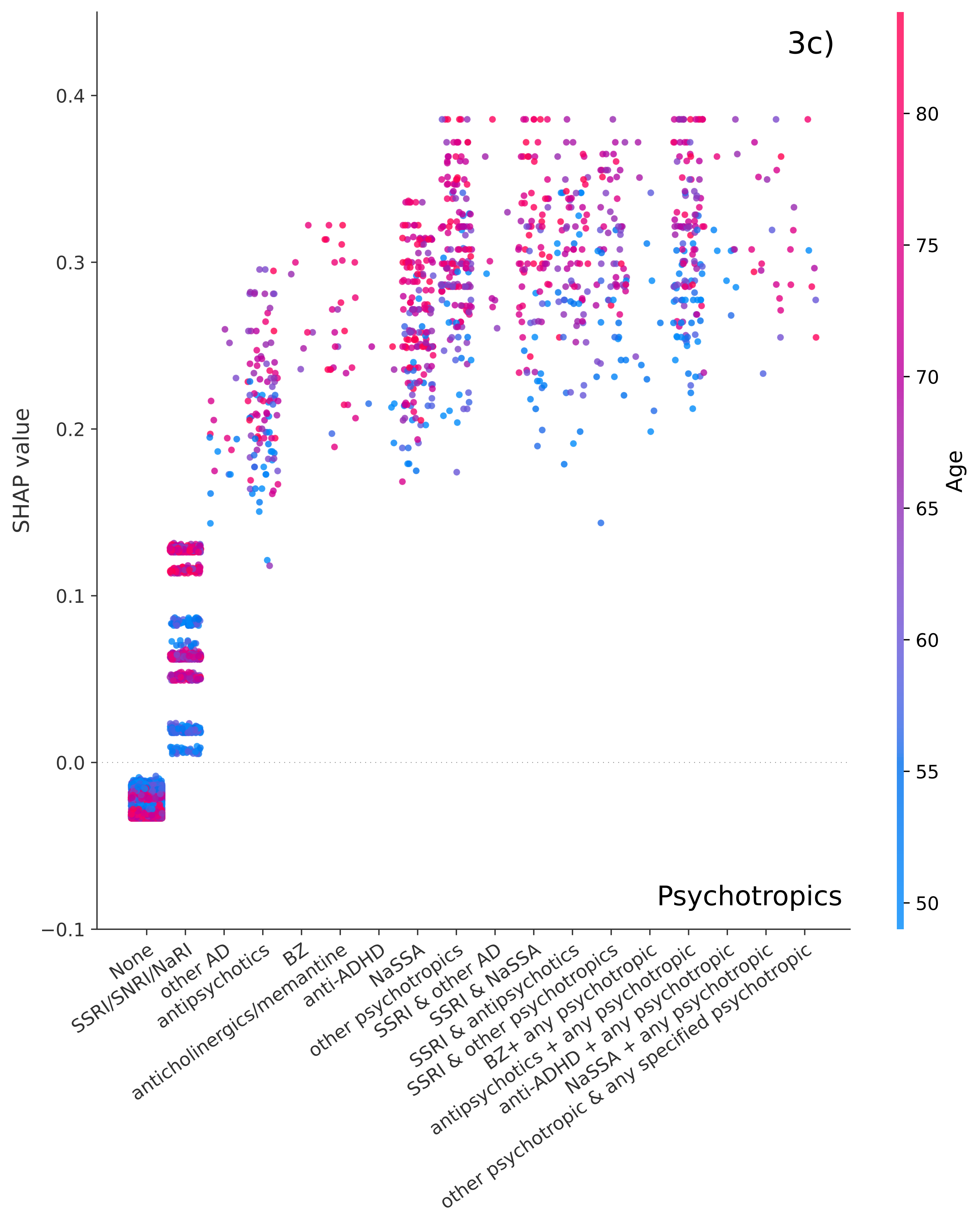
3c)
SHAP value
0.4
0.3
0.2
0.1
0.0
−0.1
Psychotropics
Age
80
75
70
65
60
55
50
None
SSRI/SNRI/NaRI
other AD
antipsychotics
BZ
anticholinergics/memantine
anti-ADHD
NaSSA
other psychotropics
SSRI & other AD
SSRI & NaSSA
SSRI & antipsychotics
SSRI & other psychotropics
BZ+ any psychotropic
antipsychotics + any psychotropic
anti-ADHD + any psychotropic
NaSSA + any psychotropic
other psychotropic & any specified psychotropic

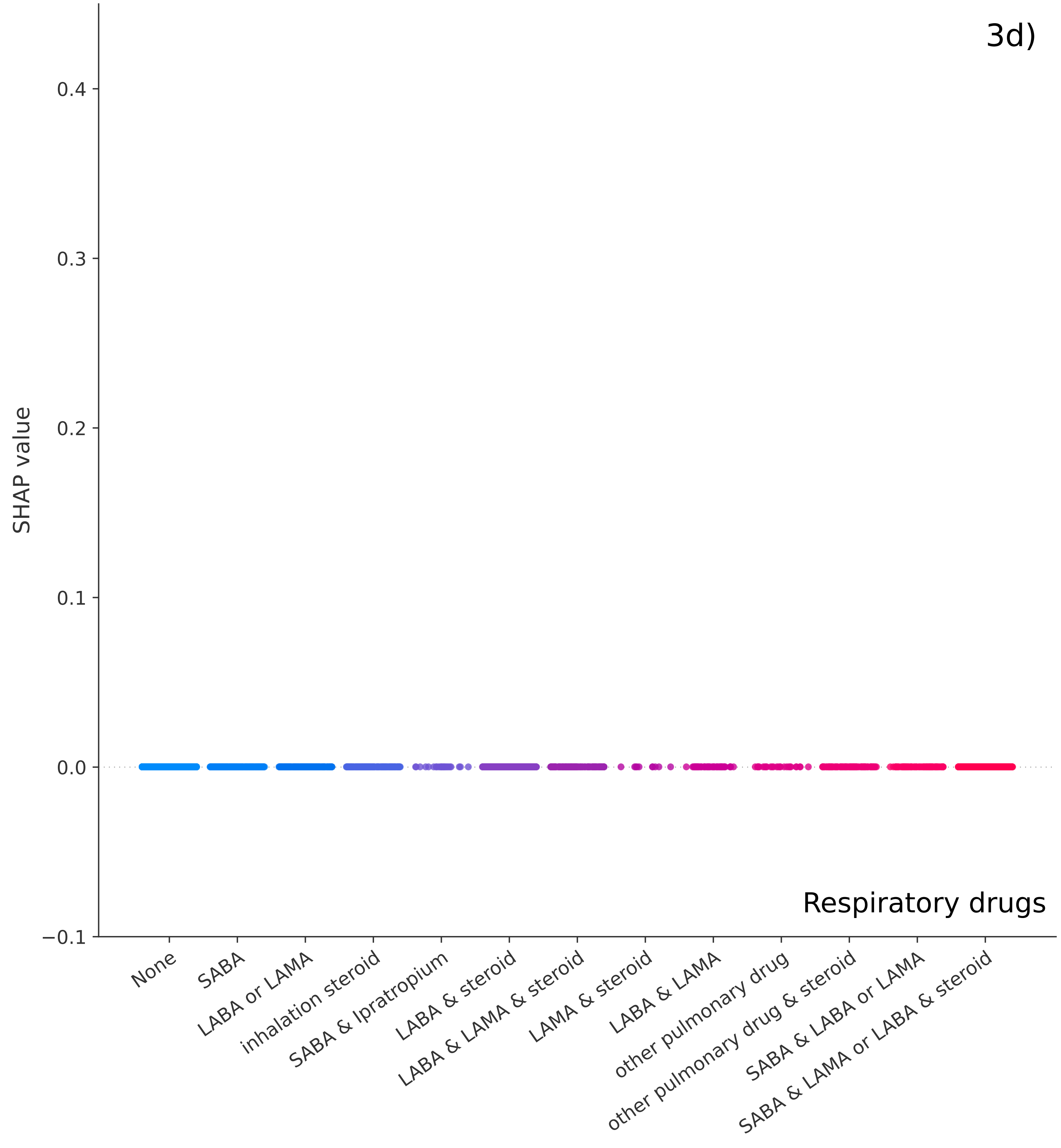
3d)
SHAP value
0.4
0.3
0.2
0.1
0.0
−0.1
Respiratory drugs
None
SABA
LABA or LAMA
inhalation steroid
SABA & Ipratropium
LABA & steroid
LABA & LAMA & steroid
LAMA & steroid
LABA & LAMA
other pulmonary drug
other pulmonary drug & steroid
SABA & LABA or LAMA
SABA & LAMA or LABA & steroid
